# Peatland inception and development across Kalimantan, Indonesia

**DOI:** 10.1038/s41598-026-35152-x

**Published:** 2026-01-20

**Authors:** Gusti Z. Anshari, Monika Ruwaimana, Rasis Putra Ritonga, Adi Gangga, Julie Loisel, Angela V. Galego-Sala, Sander van der Kaars, Nisa Novita

**Affiliations:** 1 Soil Science Department, Universitas Tanjungpura, Jalan Hadari Nawawi, Pontianak, West Kalimantan 78124 Indonesia; 2 Technobiology Faculty, Universitas Atma Jaya Yogyakarta, Jalan Babarsari No.44, Tambak Bayan, Caturtunggal, Depok, Sleman, Yogyakarta, 55281 Indonesia; 3 Yayasan Konservasi Alam Nusantara (YKAN), Graha Iskandarsyah Floor 3, Jalan Iskandarsyah Raya No. 66 C, Jakarta, 12160 Indonesia; 4 Department of Geography, University of Nevada Reno, 1664 N Virginia St, Reno, NV 89557 USA; 5 Department of Geography, University of Exeter, North Park Rd, Exeter, Ex4 4QE UK; 6 School of Earth, Atmosphere and Environment, Monash University, Melbourne, VIC 3800 Australia; 7 Magister of Environmental Science, Universitas Tanjungpura, Jalan Hadari Nawawi, Pontianak, West Kalimantan 78124 Indonesia; 8 Earth and Climate Cluster Faculty of Science, Vrije Universiteit, Amsterdam, The Netherlands

**Keywords:** Peat formation, Radiocarbon dating, Holocene, Late Pleistocene, Tropical coastal and inland peatlands, Decline in carbon sinks, Palaeoecology, Geochemistry

## Abstract

The peatlands in Kalimantan exhibit diverse geomorphological characteristics, but their initiation timing and drivers remain unclear due to limited chronological data. Using 55 radiocarbon ages and Bayesian age‒depth modeling of 15 peat cores, we reconstructed the development and carbon accumulation histories of inland and coastal peatlands in West and East Kalimantan. Coastal peat initiation occurred during the middle Holocene, coinciding with postglacial sea-level high stands, whereas inland peat formation began in the late Pleistocene. Carbon accumulation rates peaked in the middle Holocene (coastal: 63–72 g C m^−2^ yr^−1^; inland: 53–89 g C m^−2^ yr^−1^) under stable hydrological conditions but declined in the late Holocene (coastal: 49–55 g C m^−2^ yr^−1^; inland: 58–63 g C m^−2^ yr^−1^). The total decline rate was insignificant, at approximately 0.68 Mt C yr^−1^. These findings indicate that a sustained water balance is crucial for long-term peat growth and carbon sequestration and that hydrological disruption reduces the carbon storage. The current decline in the carbon sequestration capacity of drained tropical peatlands in Kalimantan is 32.4 Mt C yr^−1^ (118 Mt CO_2_-eq yr^−1^) over a 40-year period, representing approximately 47.5 times the natural decline in carbon sequestration over the past 4000 years.

**Supplementary Information:**

The online version contains supplementary material available at 10.1038/s41598-026-35152-x.

## Introduction

Tropical peatlands, renowned for their exceptionally high soil organic carbon content and abundant above-ground biomass, store more carbon per unit area than almost any other terrestrial ecosystem. In Kalimantan, Indonesia, peatlands encompass approximately 4.5 million hectares^1^ and constitute one of Southeast Asia’s most substantial carbon reservoirs. Inland peatlands in the upper Kapuas River, West Kalimantan, frequently exceed 7 m in depth, with below-ground carbon stocks of 3000–4000 Mg C per hectare being commonplace^2,3^. In certain remote upstream locations, undisturbed peat deposits reach depths of 10–18 m, harboring more than 6000 Mg C per hectare in their soils^2^. However, these extensive carbon stores have progressively been threatened in recent decades by anthropogenic disturbances, including timber extraction, land-use conversion, drainage, and recurrent fires^4–7^. Drainage, particularly, disrupts the natural hydrological regime, causing the groundwater table to drop^8–16^. This shift disrupts the peatland’s carbon balance, transforming it from a net carbon sink to a net carbon source. This transition can occur gradually through enhanced aerobic decomposition or rapidly during fire events, particularly those associated with dry seasons during El Niño years ^7,17–20^. Nevertheless, amidst these challenges, promising findings point to a recovery of the sink arise when peatlands are restored and when waterlogged conditions are reinstated^21–24^. The rewetting of peatlands previously affected by human disturbances, including drainage, has been shown to stabilizes losses of carbon and shifts the carbon balance back towards carbon accumulation^22,25–29^.

While contemporary flux measurements provide valuable short-term insights into the peatland carbon balance^30^, understanding their resilience to climatic and anthropogenic pressures requires a much longer perspective. Over millennial timescales, peat accumulation reflects the balance between organic matter inputs (from net primary productivity) and losses (through biochemical decomposition, fires, and erosion), both of which are influenced by hydroclimate, sea-level changes, and disturbance regimes. Paleoecological research offers this long-term perspective, yet previous efforts in Kalimantan have been confined to a limited number of study sites^31–35^, potentially distorting our comprehension of the long-term accumulation histories of diverse tropical peatlands across the island.

Globally, numerous studies have used basal peat radiocarbon dates to determine the timing of peatland inception and explore how carbon accumulation rates (CARs) have changed over time^36^. In temperate and boreal peatlands, the peak CAR usually occurs during the initial stages of peat development when the available peat-forming sites are rapidly filled. However, this rate gradually decreases as the domes mature, and hydrological feedback stabilizes the growth process^37–39^. In temperate Europe, North America, and South America, peat initiation commenced during the early to middle Holocene and subsequently expanded during the middle to late Holocene^40–43^. In tropical systems, accumulation histories exhibit greater variability and are frequently correlated with Holocene sea-level fluctuations, monsoon variability, and local geomorphic conditions. In Southeast Asia, coastal peatlands frequently emerged during middle Holocene sea-level high stands, while subsequent accumulation rates were influenced by shifts in the Australasian monsoon system^44,45^. Inland peat can develop independently of sea-level forcing, with initiation and growth patterns primarily governed by precipitation seasonality, groundwater availability, and catchment hydrology^46^. These findings emphasize that peat accumulation rates are not constant but can fluctuate with centennial- to millennial-scale climate oscillations, such as variations in the Intertropical Convergence Zone (ITCZ) or El Niño–Southern Oscillation dynamics, which control ground water tables in peatlands^47^.

This study advances the regional understanding of peatland dynamics by synthesizing peat cores from six coastal and nine inland peatlands in West and East Kalimantan. It incorporates 55 new radiocarbon ages from 15 peat cores, enabling the construction of a robust chronological framework for long-term peat accumulation rates. By reconstructing these temporal patterns, this study offers critical insights into the historical carbon sequestration capacity of tropical peatlands in Kalimantan.

The synthesis not only deepens the knowledge of peatland contributions to the global carbon cycle but also provides essential benchmarks for assessing recent changes in the carbon balance caused by land-use change and degradation. These long-term records form a scientifically grounded baseline for estimating carbon sequestration rates, supporting restoration target setting, climate change mitigation strategies, and regional carbon accounting frameworks.

## Methods

### Study sites

**Fig. 1:**
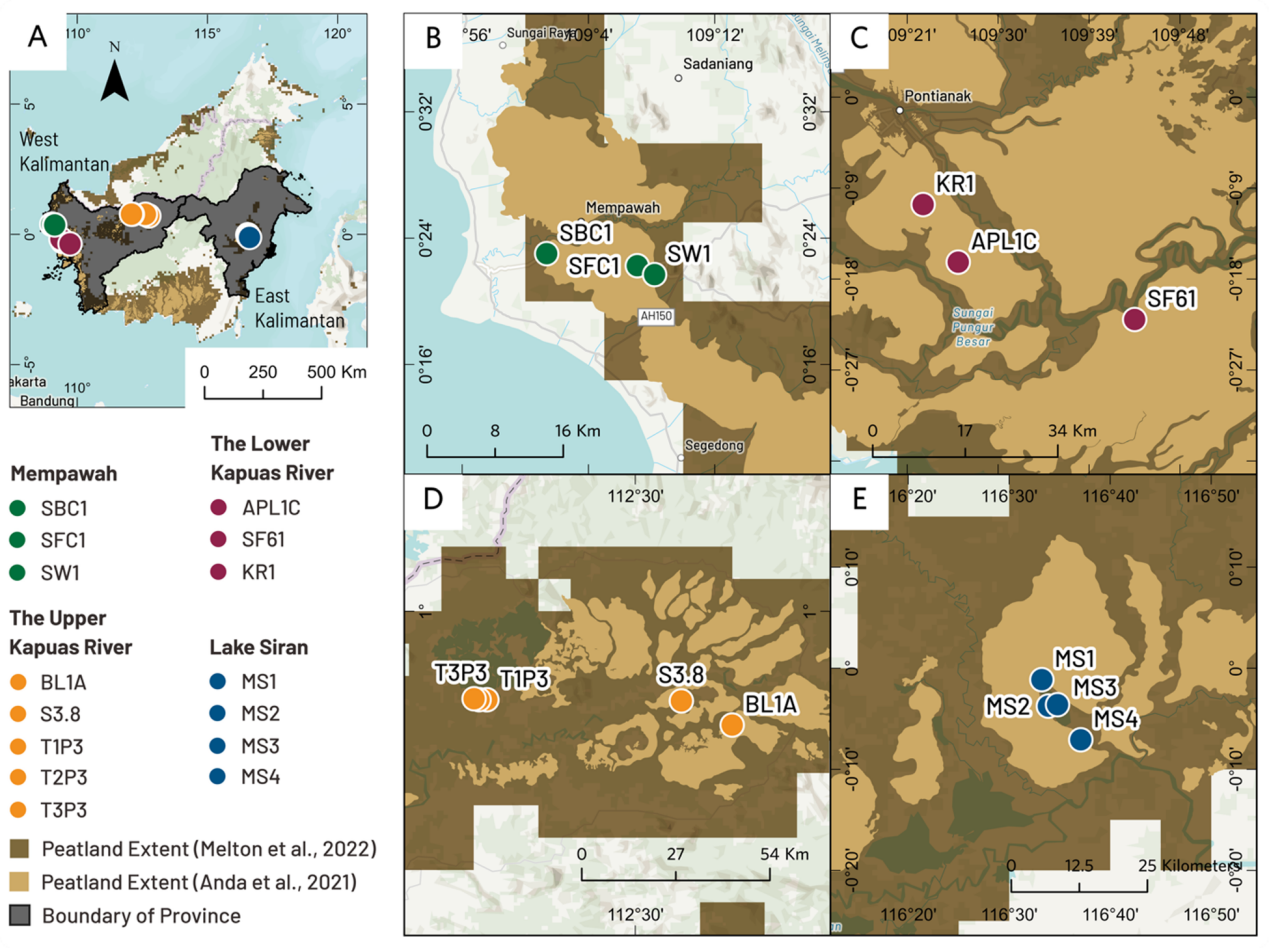
Study site locations in Kalimantan, Indonesia, including an overview of all the coring sites (**A**) and detailed maps of each region: (**B**) Mempawah District, coastal West Kalimantan (SBC1, SFC1, SW1); (**C**) lower Kapuas River, coastal West Kalimantan (APLC, SF61, KR1); (**D**) upper Kapuas River, inland West Kalimantan (BL1A, S3.8, TIP3, T2P3, T3P3); and (**E**) lake Siran, inland East Kalimantan (MS1, MS2, MS3, MS4). The peatland extent polygons in panels B, C, and E are adapted from Anda et al.^1^, and those in panel D are from Melton et al.^49^. The base map was generated in ArcGIS Pro 3.5 (Esri, https://www.esri.com/en-us/arcgis/products/arcgis-pro/overview) via the TNC World Topographic Map Reference (https://basemaps.arcgis.com/arcgis/rest/services/World_Basemap_v2/VectorTileServer). The description of the basemap is accessible at https://www.arcgis.com/home/item.html?id=a67afb8c11d840daaa27702f45a75580.

Our four study regions on the island of Kalimantan, Indonesia, span all the way from West Kalimantan Province to East Kalimantan Province (Fig. 1). We collected fifteen cores as follows: (1) three cores from Mempawah District (Coastal West Kalimantan), (2) three cores from the Lower Kapuas River (Coastal West Kalimantan), (3) five cores from Lake Sentarum National Park (Inland West Kalimantan), and (4) four cores from Lake Siran (Inland East Kalimantan). In study regions (1) and (2), peatlands are drained and/or used for agriculture, whereas in regions (3) and (4), peatlands are mostly intact. Below are descriptions of the sites by region.

1. In Mempawah District (West Kalimantan, coastal sites), we collected three peat cores: a drained shrub peatland (SBC1), a drained and fragmented secondary forest (SFC1), and an oil palm plantation (SW1). Core SBC1 is in Bukit Asam Village, the Antibar subdistrict of Mempawah. Cores SFC1 and SW1 are in Anjongan Dalam village, Anjongan subdistrict of Mempawah. These coastal peats are approximately 10 to 20 km from the Mempawah River estuary. Compared with the other two cores, the SBC1 core is positioned closer to the estuary.
2. In the lower Kapuas River (West Kalimantan coastal site), three peat cores were extracted from an oil palm (APL1C), a drained secondary forest (SF61), and a small-scale rubber plantation (KR1). These coastal peat cores are located between 30 and 50 km from the Kapuas River deltas of West Kalimantan Province. Compared with the other two cores, the SF61 core is situated at a lower elevation.
3. Peat samples were collected from the upper Kapuas River, including samples from Lake Beliung (BL1A) and Lake Siawan (S3.8), plus three cores from Lake Sentarum National Park (T1P3, T2P3, and T3P3). The peatlands in the upper Kapuas River are classified as inland peat because they are approximately 900 km from the Kapuas River delta. Access to cores BL1A and S3.8 is more challenging than access to the cores in Lake Sentarum National Park. Consequently, human activities are less common in Lakes Beliung and Siawan.
4. In East Kalimantan, we collected four inland peat cores (MS1, MS2, MS3, and MS4) from Lake Siran. Two of these cores, MS1 and MS2, were pristine undrained peat swamp forests. The MS3 core was collected from the lakebed of Lake Siran. At the time of drilling, the MS3 core was under shallow water due to a dry spell. The presence of peat under the current lake water suggests the extent of peat swamp forests in the past^48^. Finally, we collected the MS4 core from a shrub peatland near Lake Siran village. All the peats in East Kalimantan are classified as inland peat because their location is approximately 150 km away from the Mahakam River delta.

### Sample collection, laboratory methods, and radiocarbon dating

In Indonesia, peat soil (Histosol) is defined as organic soil characterized by a minimum thickness of organic matter of 50 cm^1^. To obtain peat core samples, we utilized a Russian corer to retrieve samples at 50 cm intervals until we reached the mineral substratum beneath the peat mass. The thickness of the peat soil was subsequently measured after retrieving the mineral substrates. This process involved coring the peat at 50 cm intervals, with the coring bar extending every 100 cm until reaching the mineral substratum. We classified the depth of each peatland site according to the Indonesian standard for peat thickness (SNI 7925:2019), which defines peat as organic soil characterized by a minimum thickness of organic matter of 50 cm. The standard of peat thicknesses consists of shallow (0–100 cm), moderate (100–200 cm), deep (200–300 cm), very deep (300–500 cm), extremely very deep (500–700 cm), and extraordinarily very deep (> 700 cm). We collected a single subsample to represent each 50 cm section. Each subsample measured 10 cm in length and had a volume of 100 cm^3^. We stored the peat cores in half PVC containers and wrapped them with plastic wrap for transportation.

We analyzed subsections of all cores for bulk density (BD) and total organic carbon (TOC) at 50 cm resolution for thicknesses between 0 and 200 cm, at 100 cm resolution for thicknesses between 200 and 500 cm, and at 200 cm resolution for thicknesses greater than 500 cm. The sample resolution protocol for the BD and TOC samples was modified from Kauffman et al.^50^. We assumed that the waterlogged conditions largely control the values of BD and TOC in peat. In general, the groundwater table in undrained and drained tropical peats ranges from 5–50 cm and 60–90 cm, respectively. Consequently, deeper peat layers (> 200 cm) experience more stable waterlogged conditions and are subject to lower decomposition rates. As a result, high-resolution sampling of BD and TOC in these deeper sections has a minimal impact on estimates of carbon accumulation rates, justifying a lower sampling frequency at depth.

The number of BD and TOC samples is directly proportional to the peat depths of the corresponding cores. The sample size is approximately 100 cm^3^. Owing to the inherent volatility of organic matter in peat samples, we recalculated the volume of BD samples via the water displacement method. We obtained BD values by drying our samples in a laboratory oven at 70 °C until they reached a constant weight. The TOC was measured via a Yanaco JM 1000 CN Corder.

We selected samples from the core surface, middle, and bottom sections, which extend down to the mineral substratum, for radiocarbon dating. Dry weight samples (5–10 g) of bulk peat were sent to the Waikato Radiocarbon Dating Laboratory in New Zealand for analysis. In the laboratory, visible contaminants such as living roots were removed, and the samples were chemically pretreated. The samples were washed in hot HCl, rinsed, and treated with multiple NaOH washes. The insoluble fraction was treated with hot HCl, filtered, rinsed, and dried. The laboratory reported the results of radiocarbon dates in both BP (Before Present) and calibrated date (cal. BP) using OxCal v4.4.4 (see Tables 1, 2, 3, and 4).

**Table 1:** Radiocarbon dates from coastal and drained peats in the Mempawah District, as reported by the Waikato Radiocarbon Dating Laboratory, New Zealand.

No	Core ID	Lab code	Sample depth (cm)	Age ± SD (YBP)	Age (cal. BP)
1	SFC1	Wk-56573	0–11	101 ± 0.2%	PMC*
2	SFC 1	Wk-56574	45–50	1193 ± 16	1180–1060
3	SFC1	Wk-56575	134–137	3026 ± 15	3330–3160
4	SFC1	Wk-56576	323–327	6812 ± 19	7690–7600
5	SW1	Wk-56587	0–5	112.6 ± 0.3%	PMC*
6	SW1	Wk-56588	95–100	1286 ± 16	1290–1170
7	SW1	Wk-56589	225–230	4460 ± 17	5280–4970
8	SBC1	Wk-56590	0–5	257 ± 15	420–150
9	SBC1	Wk-56591	95–100	1253 ± 16	1280–1120
10	SBC1	Wk-56592	490–497	4397 ± 16	5050–4870
11	SBC1	Wk-56593	850–853	6376 ± 19	7420–7180

* PMC = postmodern carbon. Core SFC1 is a drained and fragmented secondary forest in Anjongan Dalam Village. Core SW1 is an oil palm plantation in Anjongan Dalam Village. Core SBC1 is a shrub peat in Bukit Asam Village.

**Table 2:** Radiocarbon dates from coastal and drained peats in the lower Kapuas River basin, as reported by the Waikato Radiocarbon Dating Laboratory, New Zealand.

No	Core ID	Lab code	Sample depth (cm)	Age ± SD (YBP)	Age (cal. BP)
1	APL1C	Wk-56584	0–5	23 ± 16	250–40
2	APL1C	Wk-56585	276–284	2854 ± 15	3060–2880
3	APL1C	Wk-56585	313–317	3134 ± 17	3440–3260
4	KR1	Wk-56577	0–10	1853 ± 17	1830–1710
5	KR1	Wk-56578	140–145	3260 ± 18	3560–3400
6	KR1	Wk-56579	250–255	3759 ± 15	4230–4000
7	SF61	Wk-56580	05–10﻿	103.1 ± 0.2%	PMC
8	SF61	Wk-56581	240–250	2795 ± 15	2960–2850
9	SF61	Wk-56582	488–490	5684 ± 17	6500–6400
10	SF61	Wk-56583	678–682	5939 ± 18	6850–6670

Core APL1C is an oil palm plantation. Core KR1 is a community rubber plantation. Core SF61 is a drained and protected secondary peat forest. The surface peat core KR1 is relatively old, suggesting that some peat was lost because of anthropogenic disturbances such as fires and drainage. Like cores from Mempawah, these peats are grouped as coastal peat.

**Table 3:** Radiocarbon dates from inland and undrained peat forests in the Upper Kapuas River, as reported by the Waikato Radiocarbon Dating Laboratory, New Zealand.

No	Core ID	Lab code	Sample depth (cm)	Age ± SD (YBP)	Age (cal. BP)
1	BL1A	Wk-54999	40–50	1493 ± 20	1390–1350
2	BL1A	Wk-55000	100–105	6897 ± 22	7750–7680
3	BL1A	Wk-55001	180–190	11,930 ± 33	13,870–13,740
4	BL1A	Wk-55002	230–240	12,312 ± 34	14,448–14,090
5	BL1A	Wk-55003	390–400	14,725 ± 43	18,210–17,890
6	BL1A	Wk-55004	650–700	47,662 ± 2921	46,600
7	BL1A	Wk-55005	1250–1300	35,211 ± 538	41,350–39,350
8	BL1A	Wk-55006	1350–1400	47,300 ± 2766	46,450
9	T1P3	Wk-32491	20–25	4587 ± 59	5468–5211
10	T1P3	Wk-32485	780–785	12,074 ± 137	14,548–13,584
11	T1P3	Wk-32492	900–905	11,894 ± 60	13,918–13,575
12	T2P3	Wk-32486	10–20	3780 ± 33	4248–4079
13	T2P3	Wk-32488	681–692	12,689 ± 158	15,690–14,165
14	T3P3	Wk-32489	10–15	5716 ± 66	6668–6395
15	T3P3	Wk-32487	200–205	16,444 ± 121	19,943–19,397
16	S3.8	Wk- 32493	0–5	118.5 ± 0.3%	PMC*
17	S3.8	Wk-32494	350–355	2300 ± 30	2335–2303
18	S3.8	Wk-32495	700–705	6127 ± 62	7173–6846

Cores BL1A and S3.8 were retrieved from Lakes Beliung and Siawan, which are surrounded by undrained peat forests. Cores T1P3, T2P3, and T3P3 were undrained peat forests in Lake Sentarum National Park. The peat cores from Lakes Beliung and Siawan and Lake Sentarum National Park are grouped as inland peat. Importantly, the surface peat in cores T1P3, T2P3, and T3P3 exhibited remarkable antiquity, implying potential cessation or erosion of peatland. Frequent peat fires have been documented in the Lake Sentarum region, and it is plausible that these fires contributed to the erosion of the peat surface. In contrast, the surface peat in Lake Siawan (core S3.8) indicates a more recent or modern origin.

**Table 4:** Radiocarbon dates from inland and undrained peatlands in Lake Siran, as reported by the Waikato Radiocarbon Dating Laboratory, New Zealand.

No	Core ID	Lab code	Sample depth (cm)	Age ± SD (YBP)	Age (cal. BP)
1	MS1	Wk57329	0–5	116.7 ± 0.5%	PMC
2	MS1	Wk57330	315–320	1841 ± 17	1820–1700
3	MS1	Wk-57331	635–640	3916 ± 14	4420–4290
4	MS1	Wk-57332	900–905	4569 ± 14	5320–5280
5	MS1	Wk-57333	1185–1190	6223 ± 18	7130–7010
6	MS1	Wk-57334	1235–1240	6628 ± 16	7570–7470
7	MS2	Wk-57335	0–5	102.2 ± 0.2%	PMC
8	MS2	Wk-57336	550–555	5222 ± 14	6000–5920
9	MS2	Wk-57337	820–825	6794 ± 15	7680–7580
10	MS3	Wk-57338	0–5	2782 ± 15	2060–2840
11	MS3	Wk-57339	205–210	5348 ± 18	6210–6110
12	MS3	Wk-57340	405–410	5914 ± 15	6790–6670
13	MS3	Wk-57341	825–830	7487 ± 16	8370–8290
14	MS4	Wk-57342	0–5	105.4 ± 0.3%	PMC
15	MS4	Wk-57343	115–120	4073 ± 19	4620–4510
16	MS4	Wk-57344	190–195	4582 ± 18	5330–5280

Cores MS1, MS2, MS3, and MS4 were retrieved from Lake Siran, East Kalimantan Province. The peatland areas are predominantly submerged throughout the year and are currently less disturbed. Core MS3 was retrieved from the lakebed, and the presence of peat in this core suggests that the expansion of the lake area over the past 2000 years was driven primarily by human activities and the utilization of fire^48^.

### Data analysis

For each core, we model the age‒depth relationship via the Bacon program version 3.2.0^51^ with default parameters for the section thickness (thick = 5) and accumulation rate prior (acc. mean = 20). The IntCal20 calibration curve was applied, and for cores containing percent modern carbon (PMC) dates, a location-specific post bomb curve was included (NH3 for Northern Hemisphere sites, SH3 for Southern Hemisphere sites). The R scripts for each core are provided in Supplementary Information Table S3.

Our analysis encompasses four geological time periods: the late Pleistocene (41–11.7 kcal YBP), the early Holocene (11.7–8.2 kcal YBP), the middle Holocene (8.2–4.2 kcal YBP), and the late Holocene (4.2 kcal YBP to present). To determine the apparent carbon accumulation rates, we first calculated the rate of peat accumulation (derived from the Bayesian model in cm yr^−1^, see Fig. S1), multiplied it by peat bulk density values (g cm^−3^), and then multiplied it by the TOC (%) (see Eq. 1). To compare the simulated carbon accumulations, we used Welch’s ANOVA and Games–Howell post hoc comparisons because of the unequal number of samples and variances.

1 $$CAR\left( {g C m^{ - 2} yr^{ - 1} } \right) = D \left( {\frac{cm}{{yr}}} \right) \times BD \left( {\frac{g}{{cm^{3} }}} \right) \times \frac{TOC}{{100}} \times 10000$$

where CAR = carbon accumulation rate; D = peat accumulation; BD = bulk density; and TOC = total organic carbon.

## Results and discussion

### Peat core descriptions

#### Mempawah

We collected three peat cores from the Mempawah subdistrict. These sites include fragmented secondary peat forest (SFC1), oil palm (SW1), and shrubland (SBC1). These peatland sites have experienced drainage and frequent fires during the dry season in recent years. Cores SFC1, SW1, and SBC1 are located approximately 10–20 km from the South China Sea.

#### Secondary forest

Core SFC1 (327 cm) is classified as deep peat. The upper 0–50 cm layer was porous and slightly dry due to drainage. The 50–150 cm section contained moist peat with woody fragments. Between 150–250 cm, the peat remained moist with additional woody material, and the deepest Sect. (250–323 cm) consisted of solid peat with some woody fibers. The peat rests on a mineral substrate of clay and sand.

#### Oil Palm plantation

Core SW1 (230 cm), was also classified as “deep peat.” The near-surface peat (0–50 cm) was compact, likely due to human disturbances during oil palm harvesting and maintenance processes. The section between 50–200 cm contained some woody fragments. The mineral substrate (200–230 cm) consisted of clay and sand.

#### Shrubland

Core SBC1 (853 cm) was situated in well-drained peat. The upper section was aerobic and dry, and woody fragments were common in the middle sections. Near the base, the peat contained marine-influenced material and transitioned to hemic peat before resting on a clay substrate with sulfidic layers.

### The lower Kapuas River

The peat cores from the lower Kapuas River encompassed coastal peatlands within the newly established oil palm plantation (APL1C), the Permata Protected Forest (SF61), and the small-scale rubber plantation (KR1). Notably, all three peatland sites possess significant drainage canals that could influence the quality of the peat matrix, particularly in terms of bulk density, given the high decomposition rates and compaction associated with these areas. However, our analysis of the peat cores did not reveal any such effects. This phenomenon arises from the insufficient resolution of surface samples (ranging from 0 to 50 cm) to discern variations in bulk density values. It is hypothesized that an enhanced bulk density (BD) will be recorded at these disturbed sites if we are to utilize samples with a relatively high resolution.

#### Oil palm plantation

Core APL1C (317 cm) is classified as “very deep peat.” The upper Sect. (0–50 cm) is porous and fibrous, with compaction increasing to 100 cm. The fibrous peat continued to reach a depth of 150 cm, transitioning to more decomposed peat toward the middle and lower sections. The peat rests on a silty clay mineral substrate.

#### Permata protected forest

Core SF61 (682 cm) is classified as “extremely deep peat”. The surface Sect. (0–50 cm) was porous with small roots. Woody fragments were present throughout the core, and the peat gradually transitioned from less decomposed near the surface to more decomposed toward the base. The bottom section rested on silty material.

#### Rubber plantation

Core KR1 (255 cm) is categorized as “deep peat.” The surface peat (0–50 cm) is fibrous and contains living roots of rubber trees (*Hevea brasiliensis*). The fibrous peat extended up to 250 cm, while the underlying soft woods were present. The mineral substratum consisted of silty clay.

### The upper Kapuas River

Cores from three pristine inland peat swamp forests along the upper Kapuas River are the deepest among all the cores in this compilation. They present a significant finding, suggesting substantial carbon storage in undisturbed tropical peatland forests. These three peat swamp forests are found in Lake Beliung (core BL1A), Lake Siawan (core S3.8), and Lake Sentarum National Park (cores T1P3, T2P3 and T3P3).

#### Lake Beliung

Core BL1A (1400 cm) is categorized as “extremely very deep” peat. The site is mostly submerged except during the dry season. Woody fragments were present throughout the core, and the peat gradually transitioned from less decomposed near the surface to more compact peat toward the base. The deepest section rests on a mineral substrate composed of silts and clay.

#### Lake Siawan

Core S3.8 (705 cm) is classified as “exceptionally deep peat” from an undrained swamp. The upper section was porous and fibrous, while woody fragments were present throughout most of the core. The peat gradually became more compact toward the base, which rests on a white clay mineral substrate, possibly kaolinite.

#### Lake Sentarum National Park

Three cores were collected: T1P3 (905 cm), T2P3 (692 cm), and T3P3 (176 cm). These undrained peat forests are protected under the Ramsar Convention. The upper sections of all cores (0–50 cm) consisted mainly of sapric peat. Woody fragments were present throughout the cores, and the peat gradually became more compact toward the base. The deepest sections rested on a clay mineral substrate.

### Lake Siran

Lake Siran, in East Kalimantan, Indonesia, is part of the Mahakam River basin. The wetlands around it are crucial habitats for birds. The lake’s ecosystem is periodically flooded, and peatlands are submerged for 3–4 months, affecting both plants and animals. We collected peat cores from undrained forests, lakebeds, and shrubs. The peat cores from Lake Siran consisted of undrained forest (MS1 and MS2), peat on the lakebed (MS3), and shrubland (MS4).

#### Undrained forest

Two cores were collected from undrained peat forests: MS1 (1240 cm) and MS2 (825 cm). The upper sections were porous, while woody fragments were present throughout the cores. The peat gradually became more compact toward the base. Both cores rested on a clay mineral substrate.

#### Lakebed

Core MS3 (830 cm) was retrieved from the lakebed during a dry period. The upper section was fibrous and compact, with woody fragments present throughout the core. The peat gradually became more compact toward the base, which rests on a sandy silt mineral substrate.

#### Shrubland

Core MS4 (195 cm) is classified as “moderately deep peat”. The upper section contained sapric peat with some fibrous grass roots, whereas the middle section was compact. The peat rests on a silty clay substrate.

## Selected peat properties

The BD and TOC values in Mempawah ranged from 0.09–0.16 g cm^−3^ and from 45.13–56.52%, respectively. The average BD and TOC values in the lower Kapuas River were 0.12 g cm^−3^ and 52.05%, respectively. BD and TOC values for Lake Beliung and Lake Siawan in the upper Kapuas River peatlands ranged from 0.15 to 0.20 g cm^−3^ and 50.56 to 57.93%, respectively. The values of BD and TOC used to calculate the carbon accumulation rates in Lake Sentarum National Park (cores T1P3, T2P3, and T3P3) ranged from 0.10 to 0.20 g cm^−3^ and 50.56 to 51.56%, respectively. The BD and TOC values for cores MS1, MS2, and MS3 from Lake Siran ranged from 0.07 to 0.12 g cm^−3^ and from 37.63 to 55.03%, respectively. The BD and TOC values for core MS4 in Lake Siran were 0.07 g cm^−3^ and 52.25–54.94%, respectively. Table S2 presents the values of BD and TOC used to calculate the carbon accumulation rates.

## Radiocarbon dates

Peatlands in coastal areas of Anjongan Dalam (cores SFC1 and SW1) and Bukit Asam (core SBC1) in Mempawah District began forming in the middle Holocene. These three peatlands are also relatively young at their surfaces, indicating that peat accumulation has continued recently, although there may have been recent losses due to drainage and associated erosion and peat oxidation (as shown in Table 1). All the peats in Anjongan Dalam and Bukit Asam are currently experiencing drainage and repeated fires.

The basal ages of peatlands in the lower Kapuas River Basin are the youngest and are predominantly from the late Holocene. Only two dates (from the deepest core, SF61) belong to the middle Holocene epoch (see Table 2). The old date of the surface sample of the KR1 core most likely indicates the loss of modern peat due to agricultural practices such as clearance fires and/or rapid rates of peat subsidence due to drainage. The modern carbon ages of APL1C (oil palm) and SF61 (drained peat forest) indicate that there is potential for peat accumulation under current climates when anthropogenic disturbances are absent (see Table 2). The core of SF61 is situated at a lower elevation than the cores of APLC1 and KR1, as the core of SF61 was retrieved near the present river. We posit that clastic sedimentation was minimal and that the area was easily inundated during the period of sea level near the high stand (~ 7–6.5 ka). Rapid poor drainage near seashores facilitated peat initiation in the middle Holocene. The peats in the cores of APLC1 and KR1 commenced in the late Holocene because the sites are relatively distant from the sea, and rainfall is the primary factor controlling peat initiation. The ombrogenous peats in APLC1 and KR1 commenced after peat formed in the inundated delta of the lower Kapuas River.

The radiocarbon dates of the basal peat indicate that the inception of tropical peatlands in the upper Kapuas River basin commenced in the early late Pleistocene (see Table 3). Importantly, old ages on the surface of these peats, i.e., cores T1P3, T2P3 and T3P3, suggest erosion due to recent anthropogenic peat fires. Peats around Lake Sentarum suffer from recurrent fires associated with intensive fish harvesting in the dry season^52^. However, further research is needed to determine whether this cessation is a recent development caused by natural drying as the peat matures—reaching heights that no longer support waterlogged conditions—or whether these peatlands ceased accumulating millennia ago owing to other unidentified factors. Only surface samples from Lake Siawan (core S3.8) indicate recent peat accumulation. Similarly, younger peatlands in coastal regions of Kalimantan continue to accumulate actively (see Tables 1 and 2), particularly in areas with minimal human disturbance, highlighting the importance of conserving these systems—either as stable carbon stores in inland peatlands or as actively sequestering carbon sinks in the face of climate change.

The evidence of late Pleistocene peatland inception is restricted to samples from Lake Beliung (Core BL1A) and Lake Sentarum National Park (Cores T1P3; T2P3; T3 P3 and T4P3). The samples from Lake Siawan (Core S3.8) represent the youngest peatlands in the upper Kapuas River Basin (see Table 3). The upper Kapuas River basin presents some of the oldest tropical peatlands in Kalimantan^2,18,34^ and perhaps the oldest tropical peatland in Southeast Asia. Previous studies, such as those reported by Anshari et al.^53^ and Ruwaimana et al.^2^ revealed the inception of tropical peats in the upper Kapuas River basin in the late Pleistocene. Notably, no basal radiocarbon dates were found during the Last Glacial Maximum, between 23,000 and 19,000 years BP^54^. The basal date of core T3P3 (200–205 cm) is 16,444 ± 121 years before present (YBP), the most recent date in Table 3 that is closest to the Last Glacial Maximum (LGM). The absence of the emergence of inland peat in the upper Kapuas River basin during the Last Glacial Maximum (LGM) indicates drier climates characterized by rainfall seasonality that hindered the establishment of permanent waterlogged environments conducive to peat accumulation. The current data do not support the assertion that the late Pleistocene peats in the upper Kapuas River basin were subjected to aerobic oxidation or thermal combustion due to dry climates and peat fires during the LGM.

Table 4 presents radiocarbon dates of peats from Lake Siran in East Kalimantan. The forest peat samples (Cores MS1 and MS2) indicate that these peats commenced only in the middle Holocene and that the surface peats are substantially young. The basal peat at the MS4 site is even younger, suggesting that the inception of peatlands in the area started only in the early Holocene. The basal date from the lakebed (core MS3) also suggests an early Holocene timing of inception for this peatland. The peat is primarily composed of sapric organic matter, including decomposed woody material, indicating that it originated from forested peatlands rather than from pure sediment accumulation. Past peat fires likely contributed to forest removal and subsequent lake expansion, implying that the extent of peat forests in the late Holocene was greater than that in the current area. Cores MS1 and MS2 represent undrained peat forests.

## Variability in peat accumulation rates

**Fig. 2:**
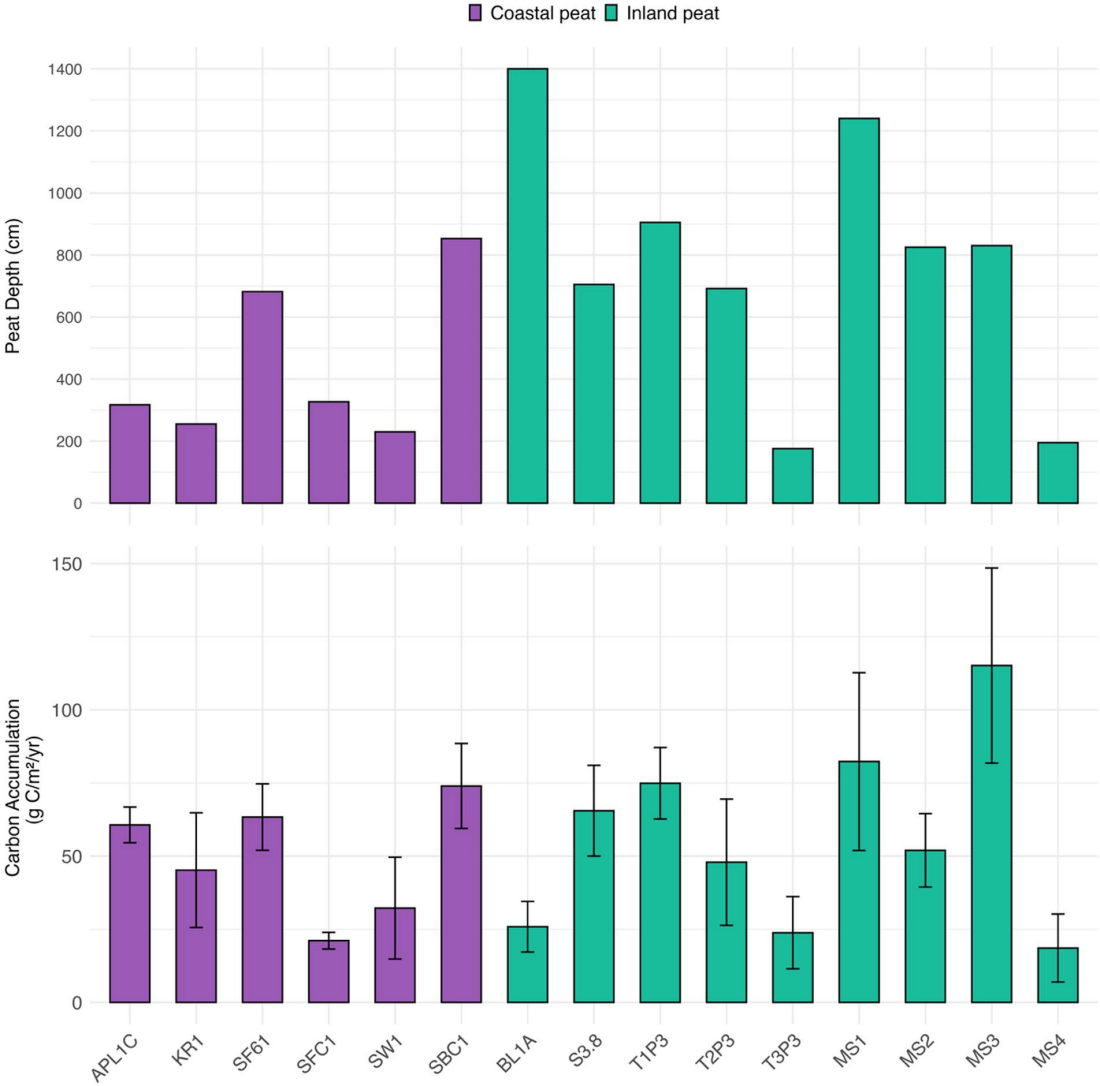
Shows the variability in peat depth during sample campaigns (top bar chart) and the mean carbon accumulation rates across 15 sites (coastal and inland peat, bottom bar chart). Carbon accumulation rates (CARs) were estimated via Bayesian age‒depth modeling with the Bacon program. We utilized RStudio version 2025.09.1 + 401, developed by Posit Software, PBC (https://posit.co), for data analysis and graph creation.

Figure 2 shows peat depth variability (top) and carbon accumulation rates (bottom) across cores. Coastal peats are shallower than inland peats. The average coastal peat depth is 444 cm, whereas the average inland peat depth is 774 cm. The deepest coastal peats are in Mempawah and the lower Kapuas River basin, reaching depths of 853 cm (SBC1 core) and 682 cm (SF61 core). The deepest inland peat is in Lake Beliung, in the upper Kapuas River, and reaches 1400 cm (BL1A core). Lake Sentarum peat depths range from 176 to 905 cm. Inland peat depths in Lake Siran range from 195 to 1240 cm. Core MS4 is the most disturbed and has the lowest depth.

Figure 2, the bottom bar chart, shows the carbon accumulation rates of the peat sample cores. In Mempawah, the shrub peat (SBC1, 74 g C m^−2^ yr^−1^) presented a higher carbon accumulation rate than did the secondary forest (SFC1, 21 g C m^−2^ yr^−1^) and the oil palm (SW1, 32 g C m^−2^ yr^−1^). The SBC1 core is situated in proximity to the coast, and its basal peat contains a portion of marine sediment. Conversely, both the SFC1 and SW1 cores are characterized by sandy substrates beneath the peat mass. These distinctions in mineral substrates suggest that the peat near the coast originated from a mangrove forest, whereas the sandy substrate indicates kerangas or heath forest. Generally, the growth rate of mangroves is greater than that of kerangas^55–57^ In Lower Kapuas, the apparent carbon accumulation rates in APL1C and SF61 (oil palm and secondary forest) were comparable (61 and 63 g C m^−2^ yr^−1^), whereas the rates in the currently smallholder rubber plantation (KR1) were lower (45 g C m^−2^ yr^−1^).

The highest rate of carbon accumulation occurred in the inland peat area (Core MS3, 115 g C m^−2^ yr^−1^) in Lake Siran. The second highest rate of apparent carbon accumulation (82 g C m^−2^ yr^−1^) was recorded in core MS1 in Lake Siran. In comparison, the highest carbon accumulation in the upper Kapuas River basin was recorded in core S3.8 (Lake Siawan, 66 g C m^−2^ yr^−1^). Like the peat in core MS1, the peat in core S3.8 appears to be actively accumulating at present. In contrast, peat in core MS3 is preserved under permanently submerged lake water. Overall, the rates of apparent carbon accumulation in coastal and inland peats are 57.0 ± 22.2 and 61.8 ± 39.3 g C m^−2^ yr^−1^, respectively (see Table S1). This estimate is consistent with that of Kurnianto et al.^58^, who reported that the carbon accumulation rates of inland and coastal tropical peat swamp forests in Southeast Asia were between 30 and 60 g C m^−2^ yr^−1^. Furthermore, the long-term median apparent carbon accumulation in tropical peatlands in Kalimantan, Sumatra, and Peninsula Malaysia was 67 g C m^−2^ yr^−1^^35,59^.

**Fig. 3:**
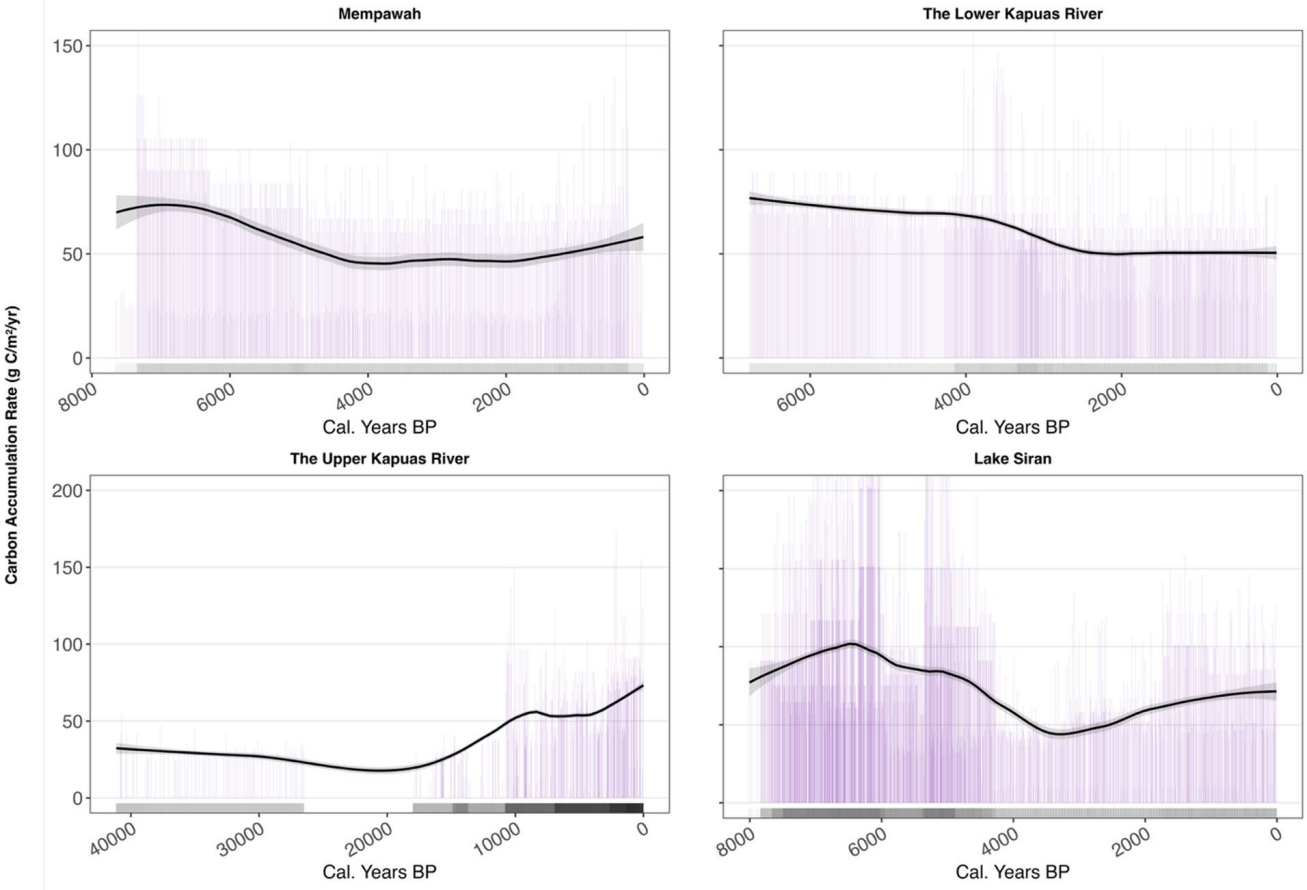
Bayesian age–depth model simulations for peat cores from Mempawah, the Lower Kapuas River, the Upper Kapuas River, and Lake Siran. The x-axis shows calendar years before present (Cal. Years BP), and the y-axis indicates modeled peat accumulation over time. Lilac bars represent posterior age probabilities for individual depth sections, whereas the black lines denote mean modeled accumulation trends with shaded 95% CIs. The horizontal bars along the x-axis represent the positions of the radiocarbon age controls used in the model; denser clusters indicate better chronological constraints and narrower uncertainty. The models reveal generally stable or slightly declining accumulation trends at coastal sites (Lower Kapuas River and Mempawah) since the middle Holocene, variable accumulation in Lake Siran, and markedly increasing accumulation rates in the Upper Kapuas River since the early Holocene. The graphs were generated via RStudio version 2025.09.1 + 401, a software product developed by Posit Software, PBC (https://posit.co).

Figure 3 shows the history of peatland inception in Kalimantan. The initiation of peatlands in the late Pleistocene was spatially restricted to the upper Kapuas River. Ruwaimana et al. and Anshari et al.^34,53^ also recorded the occurrence of peats in the late Pleistocene in the upper Kapuas River region. The occurrence of peat formation in the last ice age in the upper Kapuas River is special and might be related to the existence of depression areas, which were mostly inundated in the last glacial period, when the seasonal rainfall was able to create wetter and waterlogged conditions favorable for peat formation in the upper Kapuas River region. In other parts of Borneo, land cover in the last glacial period was probably savanna or grasslands and forests on drylands^60–63^, which were not favorable for peat formation. Figure S2 shows the distribution of the basal ages of coastal and inland peat deposits in West and East Kalimantan.

The coastal peatlands primarily began accumulating during the Holocene epoch. Notably, coastal peat exhibited substantial accumulation during the late Holocene. In contrast, the peatlands within the Permata peat forests (SF61) started accumulating in the middle Holocene. Furthermore, the carbon accumulation rates in the lower Kapuas River are notably lower than those in the upper Kapuas River.

The Bayesian-Age simulation suggests sustained peat formation from the late Pleistocene to the Holocene in the upper Kapuas River basin. The rates of peat accumulation are significantly higher in the mid-Holocene for coastal and inland peats (Fig. 3). This finding strongly indicates that both coastal and inland peats in Kalimantan play a substantial role in carbon sequestration over 40,000 years. However, this role as a carbon sink is maintained only under effective protection and sustainable management of the peat forest ecosystem. Under current anthropogenic disturbances, the environmental service of carbon sequestration by tropical peat forest ecosystems is at risk of extinction.

**Fig. 4:**
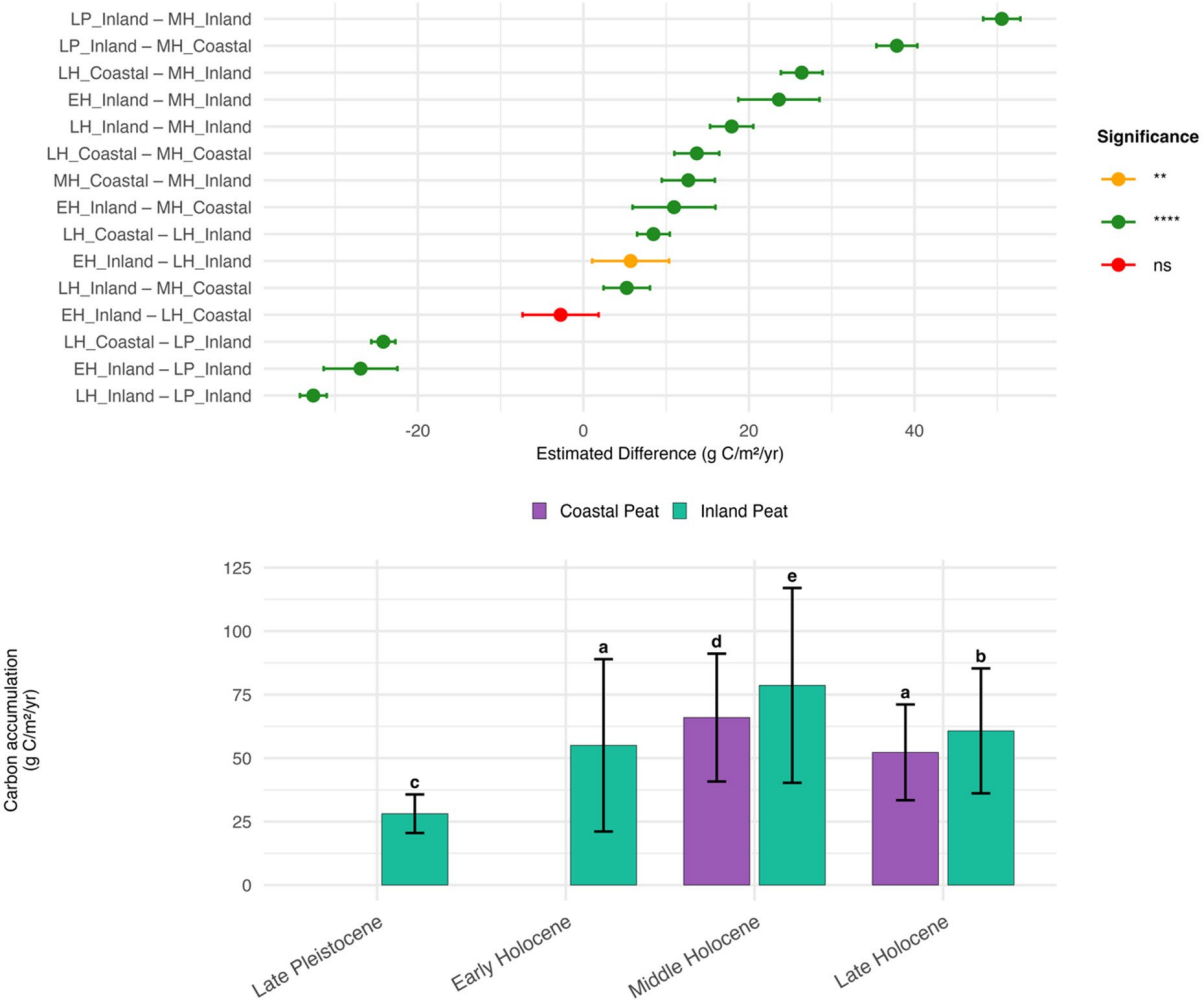
Carbon accumulation across peat types and epochs with statistical contrasts. The top panel shows pairwise differences in carbon accumulation rates (g C m⁻^2^ yr⁻^1^) between peat types and epochs based on Games–Howell post hoc tests. The green and orange dots indicate statistically significant differences (*p* < 0.05), whereas the red dot indicates a nonsignificant (ns) difference. Abbreviations: LP = late Pleistocene; EH = early Holocene; MH = middle Holocene; LH = late Holocene. The bottom panel shows the mean ± SD carbon accumulation rates (g C m⁻^2^ yr⁻^1^) for coastal (medium purple/amethyst) and inland (blue‒green/turquoise) peat types across four epochs. Letters above bars denote compact letter display (CLD) groupings; bars sharing the same letter are not significantly different (Games–Howell, α = 0.05). We used Posit Software’s RStudio version 2025.09.1 + 401 (https://posit.co) to analyze the data and generate graphs.

Figure 4 presents the results of the Welch ANOVA and Games–Howell post hoc tests on the rates of apparent carbon accumulation by geological epoch for coastal and inland peats. During the middle Holocene, inland peat carbon accumulation rates were the highest, whereas the lowest rates were recorded in inland peat during the late Pleistocene. Generally, the carbon accumulation rates in inland peat were higher than those in coastal peat during both the late and middle Holocene. Notably, the rate of carbon accumulation in coastal peat during the late Holocene was only slightly lower than that in inland peat during the early Holocene and was not significantly different.

During the middle Holocene, the combination of wet climates and rising sea levels in coastal Borneo facilitated the expansion of peatlands. For example, peat initiation occurred in the Rajang Delta, northern Borneo, at approximately 7500 YBP in clay-rich sediment areas characterized by high water tables. These favorable conditions enable vegetation growth and sustain organic matter supplies, which are essential for peat formation^64^. The rate of carbon accumulation in coastal peats was approximately 77 g C m⁻2 yr⁻1^45^. The formation of inland peat in Sebangau National Park occurred at a slow pace during the late Pleistocene. However, it experienced a peak rate of approximately 90 g C m⁻^2^ yr⁻^1^ during the early to middle Holocene. The rate subsequently declined to between 7 and 24 g C m⁻^2^ yr⁻^1^ in the late Holocene^35^. Wetter climates in the upper Kapuas River basin, West Kalimantan, drove the initiation of inland peat^65^.

## Regional implications

Our findings indicate that the development of tropical peatlands is intricate, with each peatland region in Kalimantan possessing a distinct history and set of factors influencing peat inception and growth. Peatland development is influenced by various factors, including climate, vegetation, sea level changes, tectonic movements, and the local hydrology of the site. Kessler and Jong^66^ highlight the initiation of peatlands in Borneo and indicates that it was restricted to water-logged environments, such as the area that is presently the basin of the upper Kapuas River, a region topographically depressed that is at present the floodplain of the upper reaches of the Kapuas River. Our data indicate that the climate likely sustained peatlands only in perpetual waterlogged habitats of tropical peat-forming forest trees. Continuous organic matter inputs and reduced decomposition rates facilitate peat accumulation and long-term belowground carbon storage. This idea is consistent with other studies showing that waterlogged environments create the conditions necessary for peat accumulation in the soil^64^ and subsequent belowground carbon storage in peatland ecosystems^35,44,45,67,68^.

The occurrence of tropical peat swamp forests in Borneo during the late Pleistocene is not consistent with a hypothesized savanna corridor in Sundaland during the last ice age^69^. Our findings support the contention advanced by Cannon et al.^63,70^, which posits the extensive presence of tropical forests in Borneo during the last glacial period or, at the very least, the hypothesis of the existence of forest mosaics across the landscape^71^. Having forests during the last glacial period in Sundaland would have maintained high moisture content in soils and in the lower atmosphere. In the Kapuas River Basin, it is expected that water in peatlands would have reflowed to rivers when the water level of rivers dropped during the dry season, helping regulate the hydrological cycle of the region, as is the process at present^72^. Tropical peatlands are equally important as both hydrological regulators and carbon sinks.

The presence of peat in both the late Pleistocene and the Holocene strongly indicates the resilience of tropical peat forest ecosystems to climate change^73^. The factors contributing to this high resilience include stable hydrology, which facilitates the creation of waterlogged environments, and the availability of woody organic matter for peat accumulation. However, under significant anthropogenic disturbances and climate change, preserving the function of tropical peat as a carbon sink is challenging unless nations where the remaining tropical peat exists collaborate to develop strategies and actions to achieve the sustainable goals of peatland conservation and restoration.

The rates of apparent carbon accumulation in the middle Holocene and late Holocene significantly declined (see Fig. 4). Carbon accumulation declined from the middle Holocene to the late Holocene by 13.7 ± 24.0 g C m^−2^ yr^−1^ in coastal peat and 17.9 ± 32.0 g C m^−2^ yr^−1^ in inland peat, corresponding to reductions of 20.8% ± 36.4% and 22.8% ± 40.7%, respectively. This is consistent with the findings of Sieffermann et al.^74^, who reported reduced peat accumulation rates in Central Kalimantan in the late Holocene, and with the findings of Ruwaimana et al.^18^, who reported declining rates of accumulation in coastal peatlands of West Kalimantan throughout the past four thousand years due to increased fire frequency.

The peatland area in Kalimantan spans approximately 4.5 million hectares^1^. Consequently, the long-term decline in carbon sequestration capacity over a period of 4000 years was estimated to be approximately 0.68 Mt C yr^−1^ (coastal peat ~ 0.4 and inland peat ~ 0.28 Mt C yr^−1^). The estimated area of drained peatlands in Kalimantan is 2.7 million hectares. Considering the emission factor of 12 t C ha^−1^ yr^−1^ for drained peats^10,16,75^, the conservative estimate of carbon loss solely due to drainage (excluding wildfires) is 32.4 Mt C yr^−1^ over 40 years of economic development. This figure indicates a loss of carbon sequestration capacity approximately 47.5 times greater than that sustained over the preceding 4000 years.

## Conclusions

This study synthesizes 55 new radiocarbon ages with Bayesian age–depth modeling to reconstruct peatland initiation and long-term carbon accumulation across Kalimantan. Inland peatlands in Upper Kapuas began accumulating carbon in the late Pleistocene, whereas most coastal peatlands formed in the Holocene. The rates of carbon accumulation in coastal peats in Mempawah and in the lower Kapuas River have naturally declined since the middle Holocene. Conversely, the rates of carbon accumulation of inland peat in the upper Kapuas River and Lake Siran during the middle Holocene were more rapid than those of coastal peat.

However, drainage over the past four decades has driven annual losses of ~ 32.4 Mt C (~ 118.6 Mt CO₂-eq), rapidly converting peatlands from a net sink to a major source. Undisturbed sites, both coastal and inland peats, continue to accumulate carbon, underscoring the critical need for immediate conservation and restoration to maintain their role in global climate regulation. This inland-to-coastal sequence reflects a sustained regional capacity for carbon sequestration under stable hydrological regimes.

## Supplementary Information

Below is the link to the electronic supplementary material.

Supplementary Material 1

## Data Availability

All data used in this study are available within the manuscript and its Supplementary materials.
