# Supplementary material for: Peatland inception and development across Kalimantan, Indonesia
Source: Sci Rep. 2026 Jan 20;16:5496. doi: 10.1038/s41598-026-35152-x (PMC12886863; doi:10.1038/s41598-026-35152-x)
Supplement: Supplementary file 1 — Supplementary Material 1 [file 41598_2026_35152_MOESM1_ESM.docx]

**Peatland Inception and Development Across Kalimantan, Indonesia**

**Supplementary Information**

Table S1. The average carbon accumulation rates (CARs) in the tropical peatlands of Kalimantan during the late Pleistocene to Holocene epochs.

| Type | Epoch | Research Site Location | Mean ± SD (g C m^-2^ yr^-1^) |
| --- | --- | --- | --- |
| Coastal Peat | Late Holocene (4,200 -present) | Mempawah, West Kalimantan | 49.0 ± 23.2 |
| Coastal Peat | Late Holocene (4,200 -present) | The Lower Kapuas River, West Kalimantan | 54.9 ± 13.7 |
| Coastal Peat | Middle Holocene (8,200 - 4,200 YBP) | Mempawah, West Kalimantan | 63.2 ± 29.8 |
| Coastal Peat | Middle Holocene (8,200 - 4,200 YBP) | The Lower Kapuas River, West Kalimantan | 71.6 ± 7.6 |
|  |  | The average of Coastal Peat | 57.0 ± 22.2 |
| Inland Peat | Late Holocene (4,200 -present) | The Upper Kapuas River, West Kalimantan | 63.0 ± 22.0 |
| Inland Peat | Middle Holocene (8,200 - 4,200 YBP) | The Upper Kapuas River, West Kalimantan | 53.4 ± 19.9 |
| Inland Peat | Early Holocene (11,700 - 8,200 YBP) | The Upper Kapuas River, West Kalimantan | 55.0 ± 33.9 |
| Inland Peat | Late Pleistocene (41,000 - 11,700 YBP) | The Upper Kapuas River, West Kalimantan | 28.1 ± 7.6 |
| Inland Peat | Late Holocene (4,200 -present) | Lake Siran, East Kalimantan | 58.4 ± 26.9 |
| Inland Peat | Middle Holocene (8,200 - 4,200 YBP) | Lake Siran, East Kalimantan | 89.1 ± 39.3 |
|  |  | The average of Inland Peat | 61.8 ± 34.9 |
|  | The average of coastal and inland peats | | 60.4 ± 31.8 |

Table S2. Bulk density (BD) and total organic carbon (TOC) values were used to calculate the CAR.

| Plot | Depth Interval | BD (g cm-3) | TOC (%) | Research Site location |
| --- | --- | --- | --- | --- |
| SW1 | Shallow (0 - <100 cm) | 0.16 | 45.94 | Mempawah, West Kalimantan |
| SW1 | Moderate (100 - <200 cm) | 0.10 | 51.03 | Mempawah, West Kalimantan |
| SW1 | Deep (200 - <300 m cm) | 0.10 | 56.52 | Mempawah, West Kalimantan |
| SFC1 | Shallow (0 - <100 cm) | 0.10 | 50.76 | Mempawah, West Kalimantan |
| SFC1 | Moderate (100 - <200 cm) | 0.10 | 52.68 | Mempawah, West Kalimantan |
| SFC1 | Deep (200 - <300 m cm) | 0.09 | 52.60 | Mempawah, West Kalimantan |
| SFC1 | Very deep (300 - <500 cm) | 0.11 | 50.66 | Mempawah, West Kalimantan |
| SBC1 | Shallow (0 - <100 cm) | 0.13 | 50.94 | Mempawah, West Kalimantan |
| SBC1 | Moderate (100 - <200 cm) | 0.12 | 49.06 | Mempawah, West Kalimantan |
| SBC1 | Deep (200 - <300 m cm) | 0.14 | 45.98 | Mempawah, West Kalimantan |
| SBC1 | Very deep (300 - <500 cm) | 0.13 | 46.31 | Mempawah, West Kalimantan |
| SBC1 | Extremely very deep (500 - <700 cm) | 0.11 | 45.75 | Mempawah, West Kalimantan |
| SBC1 | Extraordinarily very deep (>700 cm) | 0.14 | 45.13 | Mempawah, West Kalimantan |
| APL1C; KR2; and SF61 | Shallow (0 - <100 cm)* | 0.12 | 52.05 | The Lower Kapuas, West Kalimantan |
| APL1C; KR2; and SF61 | Moderate (100 - <200 cm)* | 0.12 | 52.05 | The Lower Kapuas, West Kalimantan |
| APL1C; KR2; and SF61 | Deep (200 - <300 m cm)* | 0.12 | 52.05 | The Lower Kapuas, West Kalimantan |
| APL1C; KR2; and SF61 | Very deep (300 - <500 cm)* | 0.12 | 52.05 | The Lower Kapuas, West Kalimantan |
| APL1C; KR2; and SF61 | Extremely very deep (500 - <700 cm)* | 0.12 | 52.05 | The Lower Kapuas, West Kalimantan |
| BL1A and S3.8 | Shallow (0 - <100 cm) | 0.11 | 57.93 | The upper Kapuas, West Kalimantan |
| BL1A and S3.8 | Moderate (100 - <200 cm) | 0.11 | 57.40 | The upper Kapuas, West Kalimantan |
| BL1A and S3.8 | Deep (200 - <300 m cm) | 0.10 | 56.77 | The upper Kapuas, West Kalimantan |
| BL1A and S3.8 | Very deep (300 - <500 cm) | 0.10 | 55.87 | The upper Kapuas, West Kalimantan |
| BL1A and S3.8 | Extremely very deep (500 - <700 cm) | 0.13 | 53.61 | The upper Kapuas, West Kalimantan |
| BL1A and S3.8 | Extraordinarily very deep (>700 cm) | 0.12 | 55.41 | The upper Kapuas, West Kalimantan |
| T1P3; T2P3 and T3P3 | Shallow (0 - <100 cm) | 0.20 | 50.65 | The upper Kapuas, West Kalimantan |
| T1P3; T2P3 and T3P3 | Moderate (100 - <200 cm) | 0.18 | 50.61 | The upper Kapuas, West Kalimantan |
| T1P3; T2P3 and T3P3 | Deep (200 - <300 m cm) | 0.16 | 50.56 | The upper Kapuas, West Kalimantan |
| T1P3; T2P3 and T3P3 | Very deep (300 - <500 cm) | 0.16 | 51.20 | The upper Kapuas, West Kalimantan |
| T1P3; T2P3 and T3P3 | Extremely very deep (500 - <700 cm) | 0.15 | 51.14 | The upper Kapuas, West Kalimantan |
| T1P3; T2P3 and T3P3 | Extraordinarily very deep (>700 cm) | 0.19 | 50.64 | The upper Kapuas, West Kalimantan |
| MS1;MS2 and MS3 | Shallow (0 - <100 cm) | 0.10 | 52.25 | Lake Siran, East Kalimantan |
| MS1;MS2 and MS3 | Moderate (100 - <200 cm) | 0.09 | 54.94 | Lake Siran, East Kalimantan |
| MS1;MS2 and MS3 | Deep (200 - <300 m cm) | 0.11 | 55.03 | Lake Siran, East Kalimantan |
| MS1;MS2 and MS3 | Very deep (300 - <500 cm) | 0.10 | 46.74 | Lake Siran, East Kalimantan |
| MS1;MS2 and MS3 | Extremely very deep (500 - <700 cm) | 0.08 | 45.55 | Lake Siran, East Kalimantan |
| MS1;MS2 and MS3 | Extraordinarily very deep (>700 cm) | 0.12 | 37.63 | Lake Siran, East Kalimantan |
| MS4 | Shallow (0 - <100 cm) | 0.07 | 52.25 | Lake Siran, East Kalimantan |
| MS4 | Moderate (100 - <200 cm) | 0.07 | 54.94 | Lake Siran, East Kalimantan |

Note: * Values of BD and TOC were estimated on the basis of published data[1–3]

Table S 3. The R programming language was used to analyze the depth‒age model of 55 radiocarbon dates obtained from 15 peat core samples located in Kalimantan. The analysis was conducted via RStudio version 2025.09.1+401 via Posit Software, PBC (https://posit.co).

| #Rbacon code for analyzing the cores  ```{bacon}  #no hiatus, default param thick=5, acc.mean=20, IntCal20, for core with PMC date postbomb curve is added. The postbomb used were depends on the location fo the core.  Bacon("APL1C")  Bacon("BL1A")  Bacon("KR1")  #one PMC date, so it requires post bomb curve 3 = NH3 (northern hemisphere 3), 5= SH3 (southern hermisphere 5)  Bacon("MS1", postbomb = 3)  Bacon("MS2", postbomb = 3)  Bacon("MS3")  Bacon("MS4", postbomb = 3)  Bacon("S3.8", postbomb = 3)  Bacon("SBC1")  Bacon("SF61", postbomb = 5)  Bacon("SFC1", postbomb = 3)  Bacon("SW1", postbomb = 3)  Bacon("T1P3")  Bacon("T2P3")  Bacon("T3P3") |
| --- |


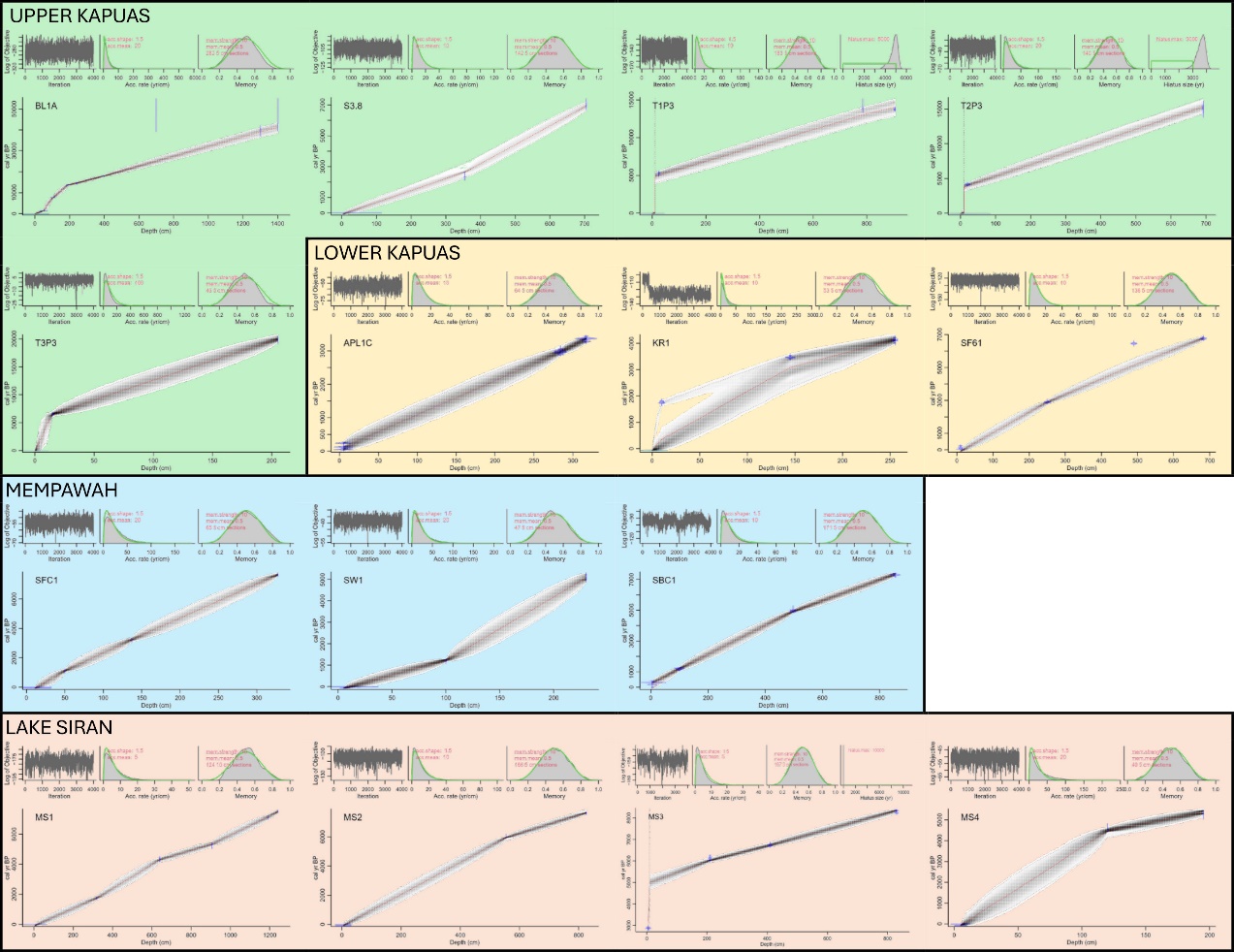


Figure S1. The results of the Bayesian Age-Depth models using the Bacon program (RStudio version 2025.09.1+401 by Posit Software, PBC, <https://posit.co>).


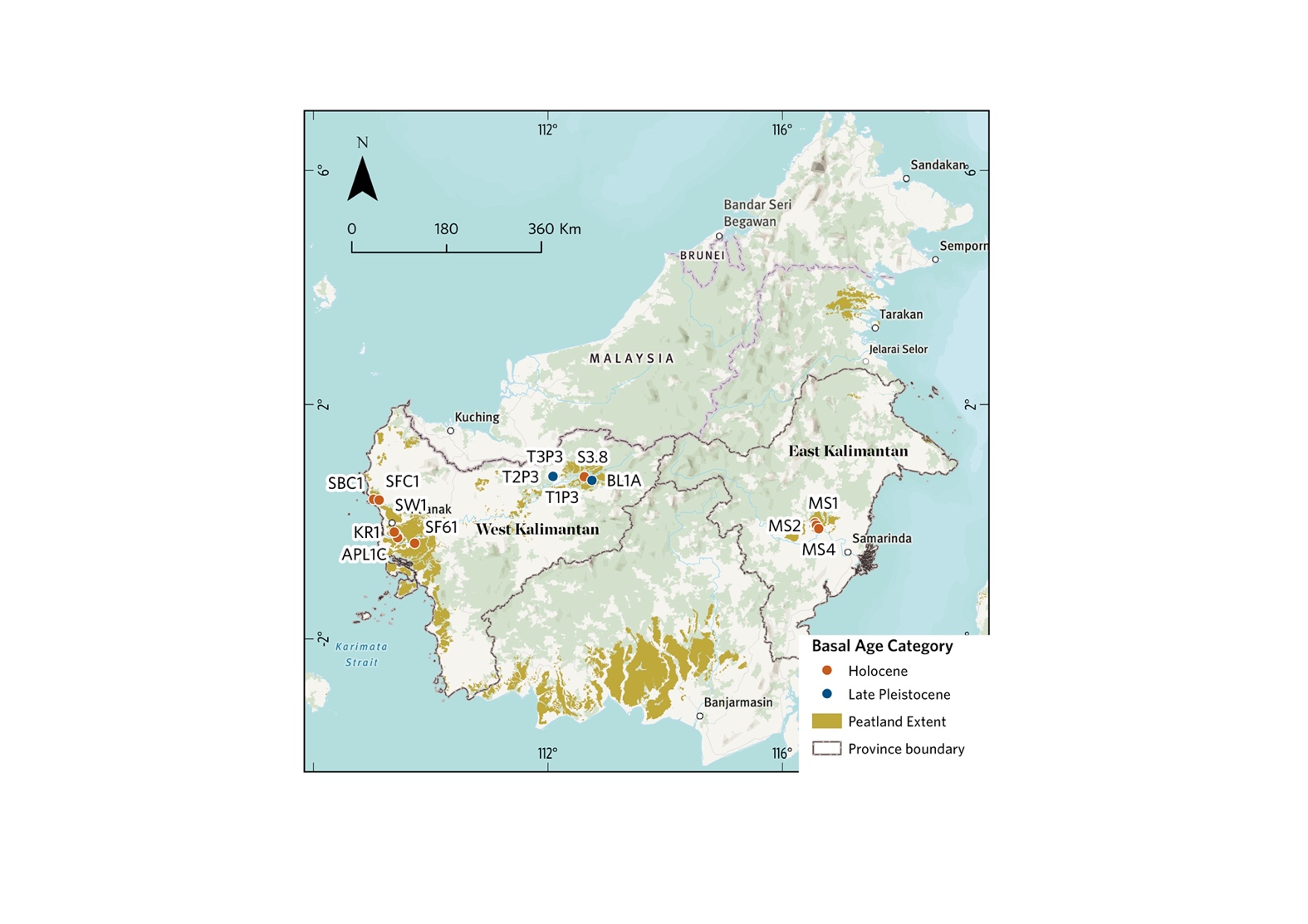


Figure S2. The basal age of coastal and inland peats in Kalimantan, showing peat initiations in the Late Pleistocene and Holocene in West and East Kalimantan, Indonesia. The base map generated in ArcGIS Pro 3.5 (Esri, https://www.esri.com/en-us/arcgis/products/arcgis-pro/overview) using the TNC World Topographic Map Reference (<https://basemaps.arcgis.com/arcgis/rest/services/World_Basemap_v2/VectorTileServer>). The description of the basemap is available at: <https://www.arcgis.com/home/item.html?id=a67afb8c11d840daaa27702f45a75580>. Peatland extent data from Anda *et al*.[4] and Melton *et al*.[5].

Reference

1. Anshari, G. *et al.* Carbon loss from a deforested and drained tropical peatland over four years as assessed from peat stratigraphy. *Catena (Amst)* **208**, 105719 (2022).

2. Hooijer, A. *et al.* Subsidence and carbon loss in drained tropical peatlands. *Biogeosciences* **9**, 1053–1071 (2012).

3. Anshari *et al.* Drainage and land use impacts on changes in selected peat properties and peat degradation in West Kalimantan Province, Indonesia. *Biogeosciences* **7**, 3403–3419 (2010).

4. Anda, M. *et al.* Revisiting tropical peatlands in Indonesia: Semidetailed mapping, extent and depth distribution assessment. *Geoderma* **402**, 1–14 (2021).

5. Melton, J. R. *et al.* A map of global peatland extent created using machine learning (Peat-ML). *Geosci Model Dev* **15**, 4709–4738 (2022).
